# Overweight and Obesity, Body Fat, Waist Circumference, and Anemia in Peruvian University Students: A Cross-Sectional Study

**DOI:** 10.1155/2021/5049037

**Published:** 2021-12-08

**Authors:** Ruth B. Quiliche Castañeda, Josué Turpo-Chaparro, Jesús Hanco Torres, Jacksaint Saintila, Percy G. Ruiz Mamani

**Affiliations:** ^1^Oficina de Nutrición, Vicerrectorado de Bienestar Universitario, Universidad Peruana Unión, Lima, Peru; ^2^Escuela de Posgrado, Universidad Peruana Unión, Lima, Peru; ^3^Escuela de Nutrición Humana, Facultad de Ciencias de la Salud, Universidad Peruana Unión, Lima, Peru; ^4^Escuela Profesional de Enfermería, Facultad de Ciencias de la Salud, Universidad Privada San Juan Bautista, Chorrillos, Peru

## Abstract

The university represents a critical space for students in terms of prevalence of malnutrition. The objective of this study was to determine the body mass index (BMI), body fat percentage (% BF), waist circumference (WC), and anemia in university students. A cross-sectional study was carried out in 2,285 university students from Lima, Peru. The sample was selected by nonprobability convenience sampling. Anthropometric data and hemoglobin levels were measured. The Chi-square test was used. The analysis of the associated factors was done using binary logistic regression. A significance level of 5% was considered. There were no significant differences between men and women in BMI (*p* > 0.05). The men presented significantly high and very high levels of % BF (*p* < 0.001). The proportion of women who presented anemia and high and very high WC was significantly higher compared to men (*p* < 0.001). Being older than 27 years (OR_B_ = 2.07; 95% CI = 1.19–3.6), being male (OR_B_ = 2.68; 95% CI = 2.02–3.55), studying at the engineering faculty (OR_B_ = 1.39; 95% CI = 1.09–1.79), having excess body fat (OR_B_ = 8.17; 95% CI = 6.13–10.87), and having an elevated WC (OR_B_ = 35.51; 95% CI = 25.06–50.33) significantly predicted overweight/obesity. The findings of this study suggest that college students, especially males and those who are not enrolled in health sciences colleges, should be a priority in healthy lifestyle interventions, particularly nutritional education programs, to reduce the prevalence of overweight and obesity.

## 1. Introduction

The university environment represents a critical space for students regarding the adoption of inappropriate eating habits and a greater risk of overweight and obesity and anemia, characterized particularly by an intake of foods rich in saturated fat and deficient in essential minerals such as iron and folic acid [1].

The prevalence of overweight and obesity has doubled since 1980 and affects almost a third of the world's population, becoming a public health problem and increasing considerably, especially among university students in developing countries [2]. In Peru, obesity is a growing problem. According to the National Institute of Health, 42.4% of young people represented by the university population have obesity [3]. Overweight and obesity represent one of the important risk factors for the development of cardiovascular diseases, insulin resistance, glucose intolerance, high blood pressure, and different types of cancer [4, 5]. These diseases contribute to reducing life expectancy by up to ten years and represent a high economic burden for society [6]. The causes of overweight and obesity are multifactorial; among them, the most prominent are inadequate dietary practices with low consumption of fruits, vegetables, whole grains, and nuts and high consumption of foods with high caloric density [7, 8]. Likewise, sedentary lifestyle and genetic and metabolic factors contribute to the appearance of the disease [9, 10].

On the other hand, another factor that is present is anemia, which is a disorder that is characterized by a hemoglobin concentration below the specific threshold, which creates an impediment to meet the oxygen demands of the tissues [11]. It is estimated that this disease affects 528.7 million women and 273.2 million children under 5 years of age in the world in 2011 [12]. In Peru, according to the National Institute of Statistics and Informatics, 20.7% of women between 15 and 49 years old had some type of anemia. 17.7% had mild anemia, 2.8% moderate anemia, and 0.2% severe anemia [13].

It is important to study young university students, because this is an important and vulnerable period for adverse behavior change related to weight and risk of anemia [14]. In Peru, some studies have been carried out regarding the prevalence of overweight and obesity [15–17]; however, few have focused on the percentage of body fat, anemia, and cardiovascular risk factors in college students. Consequently, there is an urgent need to analyze the situation in this population group. In this sense, the objective of this study was to determine the BMI, % BF, WC, and anemia in university students.

## 2. Materials and Methods

### 2.1. Design, Type of Research, and Participants

A cross-sectional study was carried out. To obtain the data, students from the first to the tenth cycle of a private university located in the eastern region of Lima, Peru, were evaluated at the beginning of the academic year. The evaluation was carried out during March 2019 (the beginning of academic cycle I). Participants were selected through intentional nonprobability sampling. Those who did not sign the informed consent and who have undergone medical treatment were not eligible to be part of the study. Before collecting the data, the participants were informed about the content of the research (nature, topic, objectives, benefits, among others). In accordance with the ethical standards for data collection (confidentiality and freedom of participation), they have also been assured that their participation was voluntary and anonymous and that they could stop filling out questionnaires and forms at any time, if they so wished. The study was approved by the Research Ethics Committee of the Universidad Peruana Unión and registered under reference number: N°0011-2019/UPeU/FCS/CIISA.

### 2.2. Anthropometric Measurements: BMI and % BF

The determinations of anthropometric and hemoglobin (Hb) measurements were carried out in the Nutritional Clinic and the Medical Center of the Universidad Peruana Unión, respectively. Weight and height were measured using a calibrated SECA 700 brand mechanical column, with scale capacity of 220 kg, and measuring range of 60 to 200 cm (SECA®, Hamburg, Germany). The BMI was calculated according to the parameters established by the Ministerio de Salud del Perú and classified as follows: underweight, <18.5 kg/m^2^; normal weight, ≥18.5 to ≤24.9 kg/m^2^; overweight, between 25.0 and 29.9 kg/m^2^; obesity ≥30 [18]. The WC was determined by means of a self-retracting metallic steel tape measured from Cescorf (Cescorf Equipamentos Para Esporte Ltda-Epp, Brazil). Abdominal obesity was considered for a WC ≥94 cm in men and ≥80 cm in women [18].

The % BF was determined using a bioelectric impedance (InBody 120, Biospace Co. Ltd., Seoul, Korea), following the recommendations proposed by the Spanish kinanthropometry group of the Spanish federation of sports medicine [19]. The measurement of the total and segmental body composition of the body (arms, legs, and trunk) using the InBody 520 system was made at 5.50 and 500 kHz. Adiposity categories were established as low-fat, normal, high-fat, and very high-fat. All procedures were performed according to the manufacturer's instructions, which are based on the work of Gallagher et al. [20].

### 2.3. Hb Concentration

Regarding the evaluation of the Hb concentration, the determination was made using the HemoCue® (HemoCue AB, Hb 201+, Angelhemo, Sweden). Once the hemoglobin value was obtained, it was compared with the cut-off points for age that are established in the Technical Health Standard for the therapeutic and preventive management of anemia in children, adolescents, and pregnant and postpartum women of the Ministry of Health of Peru. Hb levels were considered normal for men and nonpregnant women aged 15 years and over, with concentrations ≥13.0 g/dL and ≥12.0 g/dL, respectively [21].

### 2.4. Statistical Analysis

A descriptive analysis of the anthropometric and Hb parameters was carried out. Statistical hypothesis tests were carried out to compare the proportions between men and women, and between faculties. Also, the association analysis between sociodemographic characteristics and overweight/obesity was carried out. The Chi-square test was used. The association analysis between the factors associated with overweight/obesity (dependent variable) in students was evaluated using a binary logistic regression model. Age, sex, academic area, excess body fat, and elevated WC were considered as independent variables. These variables had a probability value (*p* value) lower than 0.05 in the bivariate analysis; therefore, they were included in the bivariate logistic regression analysis. These analyses were carried out considering a significance level of 5%. All analyses were done using IBM SPSS statistical software, version 24 (SPSS Inc., Chicago, IL, USA).

## 3. Results

The characteristics of the university students who participated in the study are described in Table 1. It was observed that 15.9% were under 18 years of age, the majority (61.4%) had ages ranging between 18 and 22 years, and few (4.7%) were over 27 years. 47.5% of the students came from the coast of Peru, 29.3% belonged to the mountains, 15.7% were from the jungle, and only 7.5% were foreign students residing in Peru. Regarding marital status, there was a higher representation of single students (98.4%), while a small proportion reported being married (1.6%). In relation to the distribution by academic areas, it was found that 38.5% of the students were enrolled in health sciences, 29.1% studied careers related to engineering, 16.7% studied humanities, and 15.7% studied business sciences. Fourteen professional careers were reported, where the proportions ranged from 3.6% to 13%.

Table 2 shows the descriptions about the anthropometric parameters and hemoglobin in men and women.

In Table 3, with respect to BMI, the analyses showed that there were no significant differences between the two groups, because similar proportions were observed in the different categories for BMI. Regarding %BF, higher proportions of male students with high and very high levels were observed compared to females, and these differences were significant (*p* < 0.001). Regarding WC, higher proportions of female students with high and very high levels were observed compared to males, and these differences were also significant (*p* < 0.001). In addition, significant differences (*p* < 0.001) were found in anemia between men and women, with a higher proportion of women presenting anemia compared to men.

No significant differences (*p* > 0.05) were observed because the proportions of students in the different levels of nutritional status were similar (see Table 4). Similarly, no significant differences (*p* > 0.05) were found between the proportions of students at different body fat levels. Regarding WC, there were higher proportions of students from the academic areas of health and business with high and very high levels compared to students from other academic areas, and these differences were significant (*p* < 0.001). However, obesity and high and very high fat percentage were less frequent in health science students. Likewise, there were significant differences (*p* < 0.001) in the proportions of students presenting anemia among the 4 academic areas, being more prevalent in students in the academic area of health.

With the contrast of proportions, it was found that age (>27 years) was significantly related to overweight/obesity (*p* < 0.01). No significant relationships were found between sex, marital status, and region of origin with overweight/obesity (*p* > 0.05). In turn, academic area (engineering), body fat, and waist circumference were significantly related to overweight/obesity (*p* < 0.05) (see Table 5).

In the binary logistic regression analysis, factors that were significantly related in the bivariate analysis were considered as predictors of overweight/obesity: age (0 = <27 years; 1 = ≥27 years), sex (0 = woman, 1 = man), academic area (0 = other, 1 = engineering), excess body fat (0 = no, 1 = yes), and elevated abdominal circumference (0 = no, 1 = yes). The results of the multivariate analysis (Table 6) showed that being over 27 years of age (OR_B_ = 2.07; 95% CI = 1.19–3.6), being male (OR_B_ = 2.68; 95% CI = 2.02–3.55), studying at the academic area of engineering (ORB = 1.39; 95% CI = 1.09–1.79), present excess body fat (OR_B_ = 8.17; 95% CI = 6.13–10.87), and having a high abdominal circumference (ORB = 35.51; 95% CI = 25.06–50.33) were variables that significantly predicted overweight/obesity.

## 4. Discussion

This study aimed to determine overweight, obesity, % FB, WC, and anemia in university students. The results showed that the prevalence of overweight and obesity according to BMI among Peruvian university students was similar in both sexes, although the proportion of men who were overweight and obese was slightly higher. On the other hand, binary logistic regression analyses showed that being a man represented a risk factor for excess body weight. One study found that obesity was significantly associated with males [22]. Another study reported that the risk of being overweight and obese was 3.8 times higher among male students [23]. Moreover, recent evidence from studies carried out in Peru shows that Peruvian men have a higher prevalence of overweight and obesity [24]. However, there are discrepancies with the findings reported by Soares and Barreto [25], who reported that Brazilian women are more overweight and obese than men, showing that they have 2.2 times the risk of being overweight or obese compared to men. These results are supported by other findings reported in similar studies, in which it has been observed that gender is a contributing factor in the classification of BMI in young students [26–28]. These findings were consistent with national estimates, considering the same age group for the overweight category [29, 30]. Possible justifications include the fact that university students generally have unhealthy eating habits and inadequate nutrient intake [31]. Moreover, women may have a higher concentration of leptin, an appetite regulating hormone [32, 33]. Furthermore, the prevalence was found to be similar among humanities, business, health sciences, and engineering students. Likewise, in a study that examined a convenience sample of 175 health sciences students from Lima, it was found that 28.7% were overweight and 6.3% were obese [34].

The % BF, compared with the BMI, is the best indicator for classifying overweight and obesity [35, 36]. In this study, the highest proportion of high and very high body fat percentage was observed in men compared to women, and these differences were significant. This finding is consistent with similar studies in which it has been observed that the majority of women students were healthy, while men obtained higher scores in overweight and obesity than women [37]. The possible reason for the lower % FB rate among women students could be due to their desire to be slimmer due to concerns about body image during adolescence [38], a factor that could motivate going to the gym and doing aerobic exercises to reduce weight and maintaining a specific body image, while men identify with muscle development and tend to increase food intake [39]. In addition, this could be due, at least partially, to the fact that a greater proportion of men had a higher BMI; in fact, the results of the current study showed that having excess body fat significantly predicts being overweight/obese. Body fat percentage is possibly one of the most accurate measures of obesity [40]. Available data suggest that the prevalence of obese and overweight men is much higher than that of women in some regions [41], which represents a greater risk of morbidity, considering that men accumulate more visceral fat, which is highly related to a greater cardiovascular risk. In contrast, women accumulate more subcutaneous fat before menopause, which represents a protective factor against obesity and metabolic syndrome [42].

In this study, it was observed that the proportion of women students who presented a high and very high WC level was higher compared to men, and these differences are significant (*p* < 0.001). This result is supported by findings observed in other studies, in which WC was significantly higher in women [43, 44]. However, these results are not consistent with the findings reported in a similar study [45]. WC is a better marker of abdominal fat and is an independent risk factor for cardiovascular disease [46]. When measured through imaging methods, it is directly associated with increased intraperitoneal fat [47]. WC is also considered a predictor of visceral adiposity and hypertension in young people [48]. Also, elevated waist circumference is an indicator of abdominal obesity and is the most common expression of insulin resistance and it has been identified as a powerful etiopathogenic factor for the development of type 2 diabetes mellitus and atherogenesis [49]. In the current study, having a high waist circumference is a predictor of overweight/obesity. However, an increase in WC is not necessarily associated with an increase in BMI and can be abnormal even when the BMI is within the normal range [50]. This would explain the fact that the women in our study had a lower prevalence of overweight and obesity according to BMI. Other studies suggest that the pattern of body fat distribution predicts metabolic diseases, regardless of the degree of overweight or obesity determined by BMI [51]. Our findings support the use of WC in screening as a valuable marker in identifying the risk of metabolic diseases in both men and women college students even when the BMI is normal.

Another important result to highlight in this study is that general obesity and high and very high waist circumference were very frequent among humanities, business, and engineering students. Students who were from the academic area of engineering were more likely to be overweight/obesity. These results could be due to inappropriate lifestyles, including unhealthy eating behaviors and sedentary behaviors. In fact, students enrolled in non-health-related colleges such as human nutrition, medicine, nursing, pharmacy, and others are expected to be less attentive to their eating habits [52]. In addition, the level of knowledge of these students on topics related to healthy eating and disease prevention is expected to be lower compared to health sciences students, which clearly can negatively impact their physical health [53]. Therefore, it is necessary to introduce healthy lifestyle interventions, particularly nutrition education programs, and physical activity, to improve the body weight status of university students, precisely those who are not enrolled in the faculties of health sciences.

On the other hand, in this study it was observed that the prevalence of anemia was lower among men and women. The prevalence of anemia is similar to that reported by Quispe et al. [54] in a study carried out on young people from Lima. Moreover, although the proportion of women with anemia was higher, similar results are reported in other studies [55, 56]; these results are generally explained simply by the loss of iron during menstruation. Women in this study have reached menarche. In the late teens, men quickly regain adequate iron stores, while women remain vulnerable to anemia as a result of menstrual blood loss. Therefore, they may remain anemic or become more anemic due to increased micronutrient requirements from menstruation, as well as from pregnancy and lactation [57]. However, it should be mentioned that there are factors not evaluated in this study that could explain the differences in the proportions of women university students with anemia compared to men; these factors could be ferritin, dietary factors, and higher testosterone in men [58].

### 4.1. Strengths and Limitations

The strengths of our study include a large sample size. In addition, the evaluators of the anthropometric and biochemical indicators were professional nutritionists, nurses, and specialized medical technologists. However, there are certain limitations that must be considered. For example, it did not take into account possible factors associated with the study variables such as dietary intake and physical exercise, so it is recommended to include them in future research to have more knowledge about the behavior of these variables. In addition, it is a nonprobabilistic sample made up of volunteer university students; therefore, the results of the study cannot be generalized, so it is recommended that future studies consider the calculation of the sample size as a test power established a priori and a method of random sample selection that allows having quantities of samples similar by groups.

## 5. Conclusions

It was evidenced that the proportion of men who were overweight/obesity was slightly higher compared to women. In addition, there is a higher proportion of students with high and very high levels of body fat in males. However, there was a higher percentage of women who presented high and very high WC levels compared to men. On the other hand, the studied population presented a low prevalence of anemia. Several factors associated with overweight/obesity were identified. In educational settings, particularly in universities, healthy lifestyles should be promoted, including healthy dietary practices, physical activity, and healthy behaviors in general to reduce the prevalence of overweight and obesity. Also, university teachers must actively participate in this process, because, as influencers and agents of change, they can significantly influence the lifestyles and health of students.

## Figures and Tables

**Table 1:** Sociodemographic characteristics of university students.

Variable	Total		Women		Men	
	*n*	%	*n*	%	*n*	%
Age (years)						
** **<18	363	15.9	210	16.0	153	15.7
** **18–22	1464	64.1	890	67.8	574	59.0
** **23–27	351	15.4	165	12.6	186	19.1
** **>27	107	4.7	47	3.6	60	6.2
Origin						
** **Coast	1086	47.5	638	48.6	448	46.0
** **Mountain range	670	29.3	368	28.0	302	31.0
** **Jungle	358	15.7	209	15.9	149	15.3
** **Foreign	171	7.5	97	7.4	74	7.6
Marital status						
** **Single	2249	98.4	1300	99.1	949	97.5
** **Married	36	1.6	12	0.9	24	2.5
Academic area						
** **Humanities	381	16.7	137	10.4	244	25.1
** **Business	359	15.7	216	16.5	143	14.7
** **Health	880	38.5	651	49.6	229	23.5
** **Engineering	665	29.1	308	23.5	357	36.7
Career						
** **Administration	198	8.7	122	9.3	76	7.8
** **Accounting	162	7.1	94	7.2	68	7.0
** **Education	101	4.4	74	5.6	27	2.8
** **Theology	164	7.2	0	0.0	164	16.9
** **Communication	116	5.1	63	4.8	53	5.4
** **Architecture	122	5.3	67	5.1	55	5.7
** **Food engineering	83	3.6	59	4.5	24	2.5
** **Environmental engineering	187	8.2	110	8.4	77	7.9
** **Civil engineering	172	7.5	53	4.0	119	12.2
** **Systems engineer	100	4.4	19	1.4	81	8.3
** **Human medicine	278	12.2	167	12.7	111	11.4
** **Psychology	297	13.0	234	17.8	63	6.5
** **Human nutrition	174	7.6	139	10.6	35	3.6
** **Nursing	131	5.7	111	8.5	20	2.1

*Note. n* = 2285.

**Table 2:** Anthropometric parameters and hemoglobin in university students.

Variable	Women			Men		
	M	SD	95%CI (LL–UL)	M	SD	95%CI (LL–UL)
Age (years)	20.29	3.43	(20.100–20.471)	21.03	3.98	(20.782–21.282)
Weight (kg)	57.73	9.73	(57.199–58.253)	66.87	11.53	(66.149–67.600)
Height (cm)	1.55	0.06	(1.547–1.553)	1.67	0.06	(1.663–1.671)
BMI (kg/m^2^)	24.01	3.64	(23.808–24.202)	24.02	3.65	(23.789–24.248)
% BF	32.83	4.31	(32.598–33.064)	22.00	6.27	(21.609–22.398)
WC (cm)	77.32	7.92	(76.896–77.754)	81.76	8.87	(81.201–82.318)
Hb (g/dl)	13.17	1.03	(13.116–13.228)	15.24	0.95	(15.182–15.302)

*Note. n* = 2285; BMI: body mass index; % BF: body fat percentage; WC: waist circumference; Hb: hemoglobin; CI: Confidence Interval; LL: Lower Limit; UL: Upper Limit; M: mean; SD: standard deviation.

**Table 3:** BMI, % BF, WC, and anemia in female and male university students.

Variable	Total		Women		Men		*χ*2	*p* ^*∗*^
	*n*	%	*n*	%	*n*	%		
BMI (kg/m^2^)								
** **Under weight	61	2.7	33	2.5	28	2.9	1.913	0.591
** **Normal	1487	65.1	863	65.8	624	64.1		
** **Overweight	596	26.1	342	26.1	254	26.1		
** **Obesity	141	6.2	74	5.6	67	6.9		
% BF								
** **Low	12	0.5	8	0.6	4	0.4	243.690	<0.001^*∗∗*^
** **Normal	1026	44.9	649	49.5	377	38.7		
** **High	845	37.0	564	43.0	281	28.9		
** **Very high	402	17.6	91	6.9	311	32.0		
WC								
** **Normal	1791	78.4	905	69.0	886	91.1	160.765	<0.001^*∗∗*^
** **High	357	15.6	295	22.5	62	6.4		
** **Very high	137	6.0	112	8.5	25	2.6		
Anemia								
** **Yes	180	7.9	163	12.4	17	1.7	87.756	<0.001^*∗∗*^
** **No	2105	92.1	1149	87.6	956	98.3		

*Note. n* = 2285; BMI: body mass index; % FB: body fat percentage; WC: waist circumference. ^*∗*^*p*-value. A Chi-square test (*χ*2) was used to evaluate the degree of significance of the anthropometric data and the sex of the participants. *p* represents the probability that gender is differentiated with anthropometric data and anemia. ^*∗∗*^statistical significance.

**Table 4:** BMI, % FB, WC, and anemia in humanities, business, health, and engineering students.

Variable	Humanities		Business		Health		Engineering		*χ*2	*p* ^*∗*^
	*n*	%	*n*	%	*n*	%	*n*	%		
BMI (kg/m^2^)										
** **Under weight	10	2.6	14	3.9	24	2.7	13	2.0	15.336	0.082
** **Normal	259	68.0	222	61.8	594	67.5	412	62.0		
** **Overweight	94	24.7	102	28.4	203	23.1	197	29.6		
** **Obesity	18	4.7	21	5.8	59	6.7	43	6.5		
%BF										
** **Low	2	0.5	1	0.3	3	0.3	6	0.9	16.735	0.053
** **Normal	165	43.3	162	45.1	412	46.8	287	43.2		
** **High	135	35.4	141	39.3	335	38.1	234	35.2		
** **Very high	79	20.7	55	15.3	130	14.8	138	20.8		
WC										
** **Normal	316	82.9	279	77.7	670	76.1	526	79.1	15.440	0.017^*∗∗*^
** **High	56	14.7	61	17.0	144	16.4	96	14.4		
** **Very high	9	2.4	19	5.3	66	7.5	43	6.5		
Anemia										
** **Yes	21	5.5	28	7.8	94	10.7	37	5.6	17.383	0.001^*∗∗*^
** **No	360	94.5	331	92.2	786	89.3	628	94.4		

*Note. n* = 2285; BMI: body mass index; % FB: body fat percentage; WC: waist circumference. ^*∗*^*p* value. A Chi-square test (*χ*2) was used to evaluate the degree of difference of the anthropometric data, anemia, and the academic areas of the university students. *p* represents the probability that academic areas are differentiated with anthropometric data and anemia. ^*∗∗*^Statistical significance.

**Table 5:** Analysis of the association between sociodemographic characteristics and overweight/obesity in university students.

Variable	With overweight/obesity		No overweight/obesity		*χ*2	*p* ^*∗*^
	*n*	%	*n*	%		
Age (years)						
** **<18	97	13.2	266	17.2	13.972	0.003^*∗∗*^
** **18–22	467	63.4	997	64.4		
** **23–27	126	17.1	225	14.5		
** **>27	47	6.4	60	3.9		
Sex						
** **Women	416	56.4	896	57.9	0.421	0.516
** **Men	321	43.6	652	42.1		
Marital status						
** **Single	720	97.7	1529	98.8	3.750	0.053
** **Married	17	2.3	19	1.2		
Region of origin						
** **Coast	361	49.0	725	46.8	1.017	0.797
** **Sierra	212	28.8	458	29.6		
** **Jungle	110	14.9	248	16.0		
** **Foreign	54	7.3	117	7.6		
Academic area						
** **Humanities	112	15.2	269	17.4	9.045	0.029^*∗∗*^
** **Business	123	16.7	236	15.2		
** **Health	262	35.5	618	39.9		
** **Engineering	240	32.6	425	27.5		
% BF						
** **Low	0	0.0	12	0.8	659.734	<0.001^*∗∗*^
** **Normal	88	11.9	938	60.6		
** **High	340	46.1	505	32.6		
** **Very high	309	41.9	93	6.0		
WC						
** **Normal	318	43.1	1473	95.2	815.066	<0.001^*∗∗*^
** **High	283	38.4	74	4.8		
** **Very high	136	18.5	1	0.1		

*Note. n* = 2285; %FB: body fat percentage; WC: waist circumference. ^*∗*^*p* value. A Chi-square test (*χ*2) was used to evaluate the sociodemographic, academic, and anthropometric data of the university students. *p* represents the probability that the mentioned data are associated with overweight/obesity. ^*∗∗*^statistical significance.

**Table 6:** Binary logistic regression analysis of factors associated with overweight/obesity in university students.

Variable	B	SE	Wald	*p*	OR_B_
Age (years) (0 = <27; 1 = ≥27)	0.73	0.28	6.70	0.01	2.07
Sex (0 = woman, 1 = man)	0.98	0.14	46.78	0.00	2.68
Academic area (0 = other, 1 = engineering)	0.33	0.13	6.10	0.01	1.39
Excess body fat (0 = no, 1 = yes)	2.10	0.15	207.08	0.00	8.17
Raised WC (0 = no, 1 = yes)	3.57	0.18	402.56	0.00	35.51
Constant	−3.61	0.17	470.83	0.00	0.03

*Note. n* = 2285; B: Beta coefficient, SE: standard error, *p*: probability, OR_B_: Multivariate Odds Ratio.

## Data Availability

The datasets used and analyzed during the present study are available from the corresponding author on reasonable request.
